# Reference-Based Restriction-Site-Associated DNA Sequencing Data Are Useful for Species Delineation in a Recently Diverged Asexually Reproducing Species Complex (Parmeliaceae, Ascomycota)

**DOI:** 10.3390/jof9121180

**Published:** 2023-12-09

**Authors:** Alejandrina Barcenas-Peña, Pradeep K. Divakar, Ana Crespo, Jano Nuñez-Zapata, H. Thorsten Lumbsch, Felix Grewe

**Affiliations:** 1 The Grainger Bioinformatics Center & Negaunee Integrative Research Center, Collections, Conservation and Research Division, The Field Museum, Chicago, IL 60605, USA; tlumbsch@fieldmuseum.org (H.T.L.); fgrewe@fieldmuseum.org (F.G.); 2 Department of Pharmacology, Pharmacognosy and Botany (DU Botany), Faculty of Pharmacy, Plaza de Ramón y Cajal s/n, Universidad Complutense, 28040 Madrid, Spain; pdivakar@farm.ucm.es (P.K.D.); acrespo@farm.ucm.es (A.C.); janoalexnz@gmail.com (J.N.-Z.)

**Keywords:** biodiversity, cryptic species, lichen-forming fungi, next-generation sequencing, phylogenomics, systematics, species delimitation, species complex

## Abstract

Cryptic species are common in lichen-forming fungi and have been reported from different genera in the most speciose family, Parmeliaceae. Herein, we address species delimitation in a group of mainly asexually reproducing *Parmelina* species. The morphologically distinct *P. pastillifera* was previously found nested within a morphologically circumscribed *P. tiliacea* based on several loci. However, these studies demonstrated a relatively high genetic diversity within *P. tiliacea* sensu lato. Here, we revisit the species delimitation in the group by analyzing single-nucleotide polymorphisms (SNPs) through genome-wide assessment using Restriction-Site-Associated sequencing and population genomic methods. Our data support previous studies and provide further insight into the phylogenetic relationships of the four clades found within the complex. Based on the evidence suggesting a lack of gene flow among the clades, we recognize the four clades as distinct species, *P. pastillifera* and *P. tiliacea* sensu stricto, and two new species, *P. clandestina* sp. nov. and *P. mediterranea* sp. nov.

## 1. Introduction

The delimitation of species in clades with high phenotypical plasticity and consequently a high amount of homoplasy in phenotypical datasets, such as lichen-forming fungi, has been revolutionized by using molecular data [1,2,3,4,5,6,7]. There is a growing body of evidence that cryptic species, which are distinct lineages lacking prominent distinguishing morphological or chemical characters, are common in lichen-forming fungi [2,3,8,9,10,11,12,13,14,15,16,17,18,19]. In contrast, some populations with clear morphological characteristics have remained unresolved in phylogenetic analyses based on multi-locus genetic data. This has been interpreted as a result of incomplete lineage sorting due to a recent diversification [6,20,21,22] or conspecificity of the morphotypes [23,24].

Reference-based restriction-site-associated DNA sequencing (RADseq), which generates data from thousands of loci across the genome, has been shown to be a successful and cost-effective tool for species delimitation in lichenized fungi that outperforms multi-locus approaches [25,26,27,28,29].

The genus *Parmelina,* as currently circumscribed, contains nine species [30] occurring in the Northern Hemisphere, mainly in Western Europe, the Mediterranean, and Western North America. In contrast, the sister genus *Myelochroa* is most diverse in eastern Asia [31]. The two genera are estimated to have split during the Eocene. Diversification in *Parmelina* was estimated to have occurred during the Miocene, with the ancestor of the genus probably occurring in the Turanian region and Europe or only Europe. The genus is characterized by broad, subirregular lobes with a smooth upper surface, cylindrical conidia, a white medulla, and the upper part of the inner exciple being carbonized. The latter can be seen as an amphithecial ring in a superficial view of the ascomata. The closest relative, *Myelochroa*, is mainly distinguished from *Parmelina* based on chemical characters [30,32].

The species delimitation in the genus is surprisingly complex for a relatively small genus. The sexually reproducing *Parmelina quercina* complex was shown to consist of several distinct lineages, with the Australasian clade now classified as a distantly related genus, *Austroparmelina* [33,34]. In addition, a lineage that is only distantly related to *P. tiliacea* but morphologically very similar was discovered and subsequently recognized as *P. cryptotiliacea* [9]. In contrast, the morphologically distinct *P. pastillifera* [35] was found nested within *P. tiliacea* [9]. These species reproduce mainly asexually by vegetative propagules, i.e., isidia. A subsequent study on the genetic diversity of *P. tiliacea* using three loci revealed high genetic diversity within three clusters with uneven but overlapping distributional ranges. All three clusters were shown to be present in the Canary Islands, consistent with the hypothesis that this area is a refugium for the group [36]. In another study, the nesting of *P. pastillifera* within *P. tiliacea* was interpreted as a case of speciation by split-off or budding [37], in which the origin of a new taxon does not affect the existence of the parental taxon [38].

Given the genetic diversity observed in *P. tiliacea*, which reproduces mainly asexually (cylindrical isidia), and the uncertainty of the distinction of this species from *P. pastillifera* (also asexually reproducing, isidia button-like), herein we revisit the species delimitation in the group using genome-wide assessment of single-nucleotide polymorphisms (SNPs) produced by RAD sequencing. In addition, we applied population genomic methods to measure the degree of genomic divergence and infer the levels of co-ancestry for each lineage found in our analysis.

## 2. Materials and Methods

### 2.1. Specimen Sampling

*Parmelina* samples collected in Armenia, Austria, Slovenia, France, Germany, Iran, Italy, Morocco, Norway, Portugal, Spain, Sweden, Switzerland, Tunisia, Turkey, and the United Kingdom between 2009 and 2017 were used in this study. For this study, a total of 86 representative specimens of *P. pastillifera* and *P. tiliacea* were selected, together with 5 *P. carporrhizans* and 4 *P. atricha* specimens (Table S1). Specimens were identified based on morphological characteristics [36,38]. A reference sequence of *Parmelia* sp. was downloaded from GenBank (GCA_018257885) [39] to filter for lichen fungal loci of *metagenomic* RAD sequences.

### 2.2. DNA Extraction and RAD Library Preparation

DNA extraction and RADseq libraries were constructed and sequenced at the University of Wisconsin-Madison Biotechnology Center. RADseq libraries were prepared as described in [40] using the restriction enzyme ApeKI [25] and sequenced on an NOVAseq6000Illumina Inc. (San Diego, CA, USA). The resulting RADseq data were obtained in FASTQ format.

### 2.3. RADseq Assembly

RADseq data were processed in ipyrad v.0.9.90 [41] using the bioinformatics servers at The Grainer Bioinformatics Center, Field Museum, as previously described [27]. We used the reference-based approach in ipyrad to filter for mycobiont loci, which mapped the metagenomic reads of the lichen symbiosis to a reference fungal genome of *Parmelia* sp. (GCA_018257885). We changed the parameter file in ipyrad to “gbs” and ploidy to haploid (“1”). We used a default minimum coverage of 4. Samples for which no clusters passed filtering of ipyrad were removed from the analyses.

### 2.4. Phylogenomic Analyses

We used the SNP data from the ipyrad output files, consisting of a matrix containing only variable sites, to conduct a maximum likelihood phylogenetic reconstruction with RAxML v8.2.11 [42]. The GTRCATX model was used with an ascertainment bias correction (--asc-corr=lewis). For each analysis, 100 bootstrap replicates were calculated using the fast-bootstrapping option implemented in RAxML [26,43]. The phylogenetic tree was midpoint-rooted and visualized using FigTree v1.4.3. Samples with extraordinarily long branches indicating a high sequencing error rate were removed, and RAxML was rerun with a reduced sample set. Our code for both the phylogenomic and population genomic analyses are included in Supplemental File S1.

### 2.5. Analysis of Population Structure

Differences in the population structure were calculated with a reduced dataset of 81 samples, which excluded samples of *P. carporrhizans* and *P. atricha*. A vcf output file with all variant SNPs was filtered for all SNPs with a minor allele frequency (MAF) greater than or equal to 0.05 and excluding sites on the basis of the proportion of 0.5 missing data (--max-missing 0.5) using vcftools v.0.1.15 [44]. This filtered vcf file was converted to a genlight object using the R package “vcfR”. Then, the genlight object was converted to a genind object from the R package adegenet v2.1.10 [45,46]. Subsequently, the genind object was appended with additional information settings for haploid genomes and the population memberships. The genind object and all the information enclosed were used for population genetics analysis executed in R.

We assessed the degree to which the populations are subdivided by estimating Nei’s Gst [47,48,49] and Hedrick’s G’st [48,49,50] indices. Nei’s Gst is a good measure when the mutation rate is small relative to the migration rate. Hedrick’s G’st standardizes the genetic differentiation measure and fits data with high mutation rates. Both indices were calculated as a population pairwise comparison and performed using the R package mmod v1.3.3 [51].

We chose a nonparametric approach with a Discriminant Analysis of Principal Components (DAPC) to evaluate the genetic structure of *P. pastillifera* and the *P. tiliacea* populations. DAPC performs a PCA (Principal Components Analysis) transformation of data, and then a DA (Discriminant Analysis) to separate groups. DAPC is implemented in the adegenet v2.1.10 package in R and was executed using the proportion of variance (95%) explained by the first 60 principal components. In addition, DAPC predicted the group members’ probability for each sample and displayed it in a STRUCTURE-like plot.

We used fineRADstructure [41] to estimate recently shared ancestry by patterns of genomic similarity between individuals. First, the pyRAD allele output file was converted into a fineRAD structure file using the finerad input.py script implemented in fineRADstructure tools. The dataset was reduced to contain only a minimum number of samples in a locus of four (--minsample 4). Subsequently, RADpainter and fineSTRUCTURE scripts from FineRADstructure were used to measure the population structure. A co-ancestry matrix for a haploid dataset (-p1) was generated using RADpainter, and individuals were assigned to populations using the fineSTRUCTURE Markov chain Monte Carlo (MCMC) clustering algorithm with the following arguments: -x 100,000, -z 100,000, and -y 1000. fineSTRUCTURE was also used to run a simple tree-building algorithm on the data of the co-ancestry matrix following the arguments -m T and -x 10,000. A visualization of the co-ancestry matrix, the MCMC output, and the coalescence tree were plotted out in R. 

An Analysis of Molecular Variance (AMOVA) [52] was performed to calculate the proportion of genomic variance by differences within and among clades using the R package poppr [53].

## 3. Results and Discussion

### 3.1. Assembly of RAD Sequencing

After the ipyrad assembly, filtering, and processing of all raw sequences and the reconstruction of an initial phylogenetic tree with RAxML, a final genomic dataset was considered that included 90 samples of the 95 initially processed. One sample was removed because in this sample, no clusters passed filtering in ipyrad (Pa_16634), and four samples were removed either because of contamination or misidentification that was indicated by long branches in the phylogenetic tree (Pt_17495, Pt_17340, Pt_17257, Pt_17294). The total number of filtered loci was 5189, with an average of 979.333 loci per sample (SD = 623.254), and a matrix comprised 11,895 columns (SNPs) with a missing site percentage of 79.98. For the population structure analyses, a resulting alignment matrix of 81 samples of *Parmelina pastillifera*, *P. clandestina*, *P. mediterranea*, and *P. tiliacea* s. str. comprised 8097 columns (SNPs) with a missing site percentage of 79.61, a total number of filtered loci of 5383, an average of 1013.160 loci per sample (SD = 620.025), and an average sequencing depth of 10.799 per SNP (SD = 5.742). 

### 3.2. Phylogenomic Analyses 

**Figure 1:**
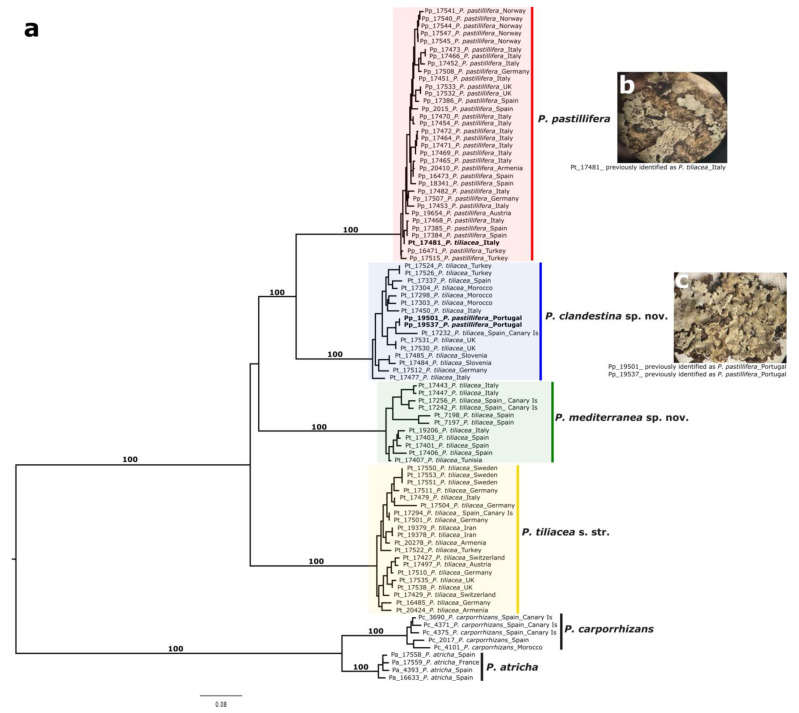
Phylogenetic tree inferred from *Parmelina pastillifera*–*tiliacea* complex RADseq data. (**a**) Maximum-likelihood phylogenetic reconstruction of *Parmelina pastillifera*–*tiliacea* complex based on concatenated DNA sequences of 5189 loci. Bootstrap values > 75 are indicated on main branches. Taxon labels include the sample’s identification before this and country of collection. (**b**,**c**) Taxa in bold and pictures highlight three misidentified specimens.

We identified six well-supported clades after conducting a phylogenetic analysis of 90 *Parmelina* samples using RADseq data (Figure 1a). Two clades comprised samples of *Parmelina carporrhizans* and *P. atricha*, respectively. Another clade consisted of samples of *P. pastillifera*, while the samples of *P. tiliacea* were clustered into three separate clades, recognized below as *P. tiliacea*, *P. clandestina*, and *P. mediterranea*. The clade here identified as *P. tiliacea* s. str. includes the epitypus specimen of the species (MAF-Lich 16485). The analysis also revealed three misidentified specimens. “Pt_174881_*P. tiliacea*_Italy” was clustered within the *P. pastillifera* clade. After reviewing this specimen, we could identify the common *P. pastillifera* character of button-like isidia (Figure 1b). In addition, the specimens “Pp_19501_*P. pastillifera*_Portugal” and “Pp_16537_*P. pastillifera*_Portugal” clustered within the *P. clandestina* clade. After reviewing these specimens, we could identify cylindrical isidia, which are commonly observed in the taxa of the *P. tiliacea* sensu lato clades (Figure 1c).

Previous studies on the species delimitation of the *Parmelina pastillifera–tiliacea* complex using nuclear ITS and mitochondrial LSU rDNA showed that *P. pastillifera* is nested within *P. tiliacea* [9,35]. A later multi-locus study, including the nuclear EF1-α marker, confirmed the genetic diversity of *P. tiliacea* sensu lato [36]. The nesting of *P. pastillifera* within *P. tiliacea* sensu lato was interpreted as a case of speciation by split-off [37,38]. However, studies based on multi-locus markers were insufficient to resolve the relationship of this group. Using reference-based RAD sequencing as a reduced genome representation method, we sequenced thousands of loci over the genome [25,28]. Unlike single-marker approaches, with RADseq, we obtained sufficient sequenced data to reconstruct a robust topology of this species complex: a clear separation of the morphologically distinct *P. pastillifera* and three cryptic lineages in *P. tiliacea*.

### 3.3. Analysis of Population Structure

For the population structure analyses of the *Parmelina pastillifera–tiliacea* complex, we reduced the RAD dataset to the specimens of the *Parmelina pastillifera*, *P. clandestina*, *P. mediterranea*, and *P. tiliacea* s. str. clades. The initial SNP matrix of 8097 columns (SNPs) was filtered for SNPs with an MAF greater than 0.05 and excluding sites on the basis of the proportion of 0.5 missing data (--max-missing 0.5). 

Nei’s Gst and Hedrick’s G’st were calculated to assess the genetic differentiation of the four species (Table 1). The differentiation measures for Nei’s Gst/Hedrick’s G’st were 0.73/0.91 between *P. clandestina* and *P. mediterranea*, 0.78/0.94 between *P. clandestina* and *P. tiliacea* s. str., and 0.77/0.93 between *P. mediterranea* and *P. tiliacea* s. str. As most G’st indices tend towards 1, the four species have isolated genomes.

**Figure 2:**
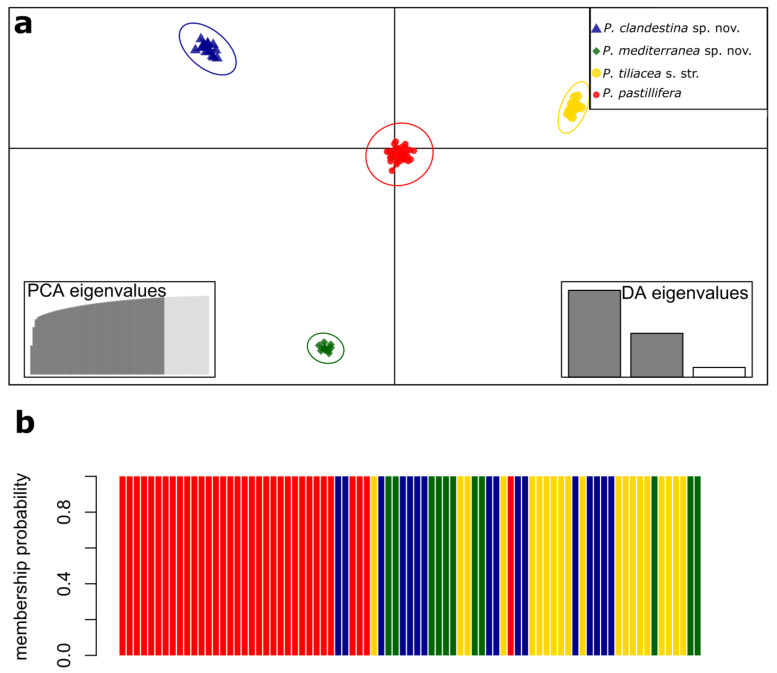
Discriminant Analysis of Principal Components (DAPC) of samples of the *Parmelina pastillifera*–*tiliacea* complex. (**a**) Scatterplot for discriminant functions. Individuals and groups are represented by dots and inertia ellipses colored as in Figure 1. The bottom-left inset graph shows the cumulative variance explained by PCA eigenvalues; dark-gray bars indicate the first 60 PCs retained. The bottom-right inset graph of the linear Discriminant Analysis (DA) eigenvalues displays the proportion of genetic variation explained by each discriminant function; dark-gray bars highlight the first two discriminant functions shown in the main scatterplot; (**b**) barplot with assigned membership probabilities. Each bar represents an individual. The colors correspond to the ones used in Figure 1.

In addition, the DAPC revealed genomic separation among the samples of the four clades (Figure 2a). Furthermore, the DAPC showed a clear distinction between the four species, as evidenced by the group members’ probability, which indicated a 100% probability of each sample belonging to its respective clade (Figure 2b).

**Figure 3:**
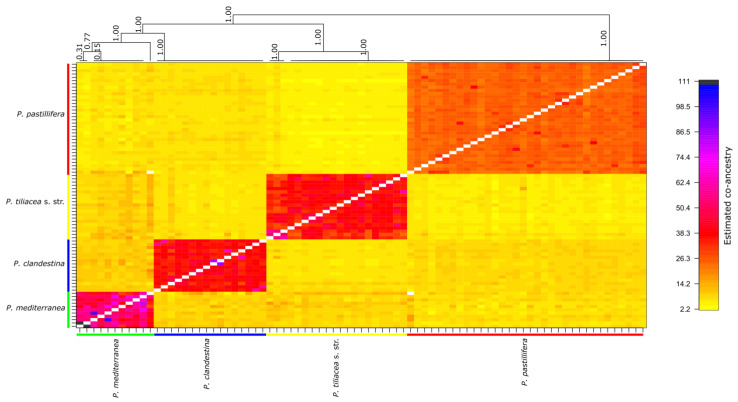
Clustered fineRADstructure co-ancestry matrix of samples of the *Parmelina pastillifera*–*tiliacea* complex. The top tree shows the population structure of the samples according to the co-ancestry matrix. Four major clades corresponding to *P. mediterranea*, *P. clandestina*, *P. tiliacea* s. str., and *P. pastillifera*. The four orange-red diagonal blocks in the co-ancestry matrix indicate that samples within the four species share more co-ancestry with each other than among species. Small black and dark-blue blocks in the *P. mediterranea* clade indicate closely related samples. The first sample pair (Pt_17256 and Pt_17242) were collected at distinct localities in the Canary Islands and the second sample pair (Pt_7197 and Pt_7198), potentially clones, were collected in Spain at the same locality.

The fineRADstructure analysis revealed that the four species correspond to the four clades identified in the phylogenetic tree. The clustering indicates a higher shared co-ancestry within each species than among them (Figure 3). Also, in the *P. mediterranea* cluster, some samples showed a very high level of co-ancestry: Pt_7197 and Pt_7198 (dark blue), which were collected at the same locality in Spain, and Pt_17256 and Pt_17242 (small black blocks), which were collected in the Canary Islands but not at the same locality. The samples also clustered in the phylogenetic tree (Figure 1). This high level of co-ancestry could indicate that these samples of *P. mediterranea* are indeed clones, which is likely when they were collected at the same locality, showing a potential case of long-distance dispersal in the Canary Islands.

The AMOVA results indicate that around ~95% of the genomic variance is due to clade variation (Table 2), solidifying a delineation of the four species of the *Parmelina pastillifera–tiliacea* complex.

All population genomic methods confirmed a high degree of genomic divergence among these clades of the complex and supported the interpretation of these clades as distinct lineages. Consequently, we recognize four species in the complex, two of which—*P. mediterranea* and *P. clandestina*—we describe as new species.

Cryptic species are common in lichen-forming fungi. RADseq, which has successfully resolved other morphologically challenging lichen groups [25,26,27,28], showed similar success in this study.

### 3.4. Taxonomy

**Figure 4:**
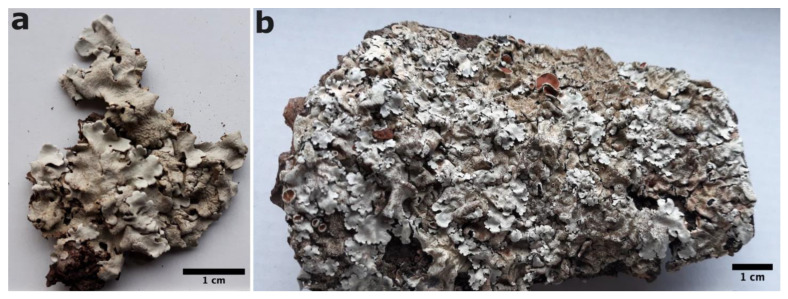
New species of *Parmelina*. (**a**) Habitus of *P. clandestina* sp. nov. (type specimen); (**b**) habitus of *P. mediterranea* sp. nov. (type specimen).

*Parmelina clandestina* Barcenas-Peña, Divakar, A. Crespo, Nuñez-Zapata, Lumbsch & Grewe sp. nov. (Figure 4a).

MycoBank: MB850743

Diagnosis: Thallus foliose pale-gray, maculate, usually pruinose, isidia cylindrical, white medulla, lower surface black, apothecia and pycnidia infrequent. Upper cortex K+ (yellow); medulla K-, C+ (red), KC+ (red), P-. Contains atranorin and lecanoric acid. Differs from morphologically similar species; *P. tiliacea* s. str. in genome data of single-nucleotide polymorphisms (SNPs) produced by RAD sequencing.

Type: MOROCCO. Ifrane, Foret Sidi, 33°37′38″ N, 05°19′51″ W, 1243 m, on *Quercus ilex*, 12 February 2011, *A. Agudo* (MAF-Lich 17298—holotype).

Etymology: the epithet refers to the enigmatic and difficult-to-detect properties of the new species.

Description: Thallus appressed to bark, pale mineral gray to mineral gray; lobes irregularly branched, sublinear to elongate, often imbricate, with rounded apices, 2–7 mm wide, margins more or less crenate and wavy, not ciliate; upper surface more or less shiny, maculate, usually pruinose, irregularly fissured, densely isidiate; white medulla; lower surface black with brown border, moderate-to-high density of black rhizines, simple, and 1–2 mm long. Isidia cylindrical, short 0.5–1.5 mm, frequently branched, usually with blackened tips. Apothecia infrequent, appressed, up to 4 mm in diameter. Asci with eight elongated ascospores, 9–13 × 5–7 μm. Pycnidia infrequent.

Chemistry: upper cortex K+ (yellow); medulla K-, C+ (red), KC+ (red), P-. Contains atranorin and lecanoric acid.

Distribution: Europe, so far observed in Germany, Italy, Morocco, Portugal, Slovenia, Spain, Turkey, and the UK.

Notes: The new species is morphologically cryptic and difficult to recognize in the field. However, in the phylogenetic tree (Figure 1), it forms a strongly supported sister relationship with *P. pastillifera*. It is sympatric with the phenotypically similar species *P. tiliacea* s. str. It can only be segregated using genetic data, and thus DNA sequencing is required to identify this species. In the field, it is easily confused with *P. tiliacea* s. str. Thus, we recommend using the term “*Parmelina tiliacea* aggregate” for field studies. 

Additional specimens examined: GERMANY. Bavaria, Oberbayern, 47°42′33″ N, 11°43′40″ E, 735 m, on *Tilia platyphyllos*, 12 September 2009, W.V. Brackel, MAF-Lich 17512; ITALY. Friuli-Venezia Giulia, Camporosso, 46°30′45″ N, 13°32′00″ E, 833 m, on *Sorbus*, 28 June 2010, J. Nuñez-Zapata et al., MAF-Lich 17477. Toscana, Monte Amiatta, 42°53′48″ N, 11°33′11″ E, 1308 m, on *Fagus sylvatica*, 6 June 2010, J. Nuñez-Zapata et al., MAF-Lich 17450; MOROCCO. Ifrane, Foret Sidi, 33°37′38″ N, 05°19′51″ W, 1243 m, on *Quercus ilex*, 12 February 2011, *A. Agudo*, MAF-Lich 17303, MAF-Lich 17304; PORTUGAL. Minho, Porto Ribeiro, 42°02′23″ N, 08°11′49″ W, 876 m, 8 September 2014, *A. Benavent*, MAF-Lich 19501. Viana do Castelo, 42°02′22″ N, 08°11′41″ W, 878m, on *Betula pubescens*, 9 September 2014, *C. G. Boluda*, MAF-Lich 19537; SLOVENIA. Bled, 46°22′08″ N, 14°06′28″ E, 475 m, on *Pinus*, 8 May 2010, J. Nuñez-Zapata, MAF-Lich 17484, MAF-Lich 17485; SPAIN. Caceres, Parque Nacional de Monfragüe, 39°42′48″ N, 05°44′20″ W, 603 m, on *Quercus*, 28 October 2010, A. Crespo et al., MAF-Lich 17337, Canary Islands, La Palma, 28°44′33″ N, 15°49′37″ W, 1993 m, on rock, 25 June 2009, A. Crespo et al., MAF-Lich 17232; TURKEY. Eskisehir, Sivirihisar Mountains, 39°25′ N, 31°40′ E, 1040 m, on volcanic rock, 7 November 2010, M. Candan, MAF-Lich 17524, MAF-Lich 17526; UK, Wales, road A5, 53°02′25″ N, 03°37′59″ W, 320 m, on *Acer*, 19 November 2010, J. Nuñez-Zapata & C. Ruibal, MAF-Lich 17530, MAF-Lich 17531.

*Parmelina mediterranea* Barcenas-Peña, Divakar, A. Crespo, Nuñez-Zapata, Lumbsch & Grewe sp. nov. (Figure 4b).

MycoBank: MB850744

Diagnosis: Thallus foliose pale-gray, maculate, usually pruinose, isidia cylindrical, white medulla, lower surface black, apothecia and pycnidia infrequent. Upper cortex K+ (yellow); medulla K-, C+ (red), KC+ (red), P-. Contains atranorin and lecanoric acid. Differs from morphologically similar species *P. tiliacea* s.tr. and *P. clandestina* in genome data of single-nucleotide polymorphisms (SNPs) produced by RAD sequencing.

Type: SPAIN. Mallorca, Municipio de Benyalbufar, Finca de Planicie, 39°40′15″ N, 02°30′34″ E, 460 m, on *Quercus ilex*, 27 November 2009, A. Crespo, P.K. Divakar & J. Nuñez-Zapata (MAF-Lich 17403—holotype). 

Etymology: the epithet refers to the geographical range of the new species, which mainly occurs in the Mediterranean region.

Description: Thallus appressed to bark, pale mineral gray to mineral gray; lobes irregularly branched, sublinear to elongate, often imbricate, with rounded apices, 2–7 mm wide, margins more or less crenate and wavy, not ciliate; upper surface more or less shiny, maculate, usually pruinose, irregularly fissured, densely isidiate; white medulla; lower surface black with brown border, moderate-to-high density of black rhizines, simple, and 1–2 mm long. Isidia cylindrical, short 0.5–1.5 mm, frequently branched, usually with blackened tips. Apothecia infrequent, appressed, up to 4 mm in diameter. Asci with eight elongated ascospores, 9–13 × 5–7 μm. Pycnidia not frequent.

Chemistry: upper cortex K+ (yellow); medulla K-, C+ (red), KC+ (red), P-. Contains atranorin and lecanoric acid.

Distribution: Mediterranean region, so far observed in Italy, Spain (including the Canary Islands), and Tunisia.

Notes: *Parmelina mediterranea* is a phenotypically cryptic species and difficult to recognize in the field. In the phylogenetic tree (Figure 1), it is clustered in the *P. tiliacea–P. pastillifera* clade with uncertain phylogenetic relationships. It is sympatric with the phenotypically similar species *P. tiliacea* s. str. and *P. clandestina*. This new species can only be identified with genetic data. Thus, we recommend using the term “*Parmelina tiliacea* aggregate” for field studies.

Additional specimens examined: ITALY. Sicilia, Santuario de Gibilmanna, Cefalu, 37°59′ N, 14°01′ E, 792 m, on *Quercus*, 7 September 2013, C. Ruibal & C. Galan, MAF-Lich 19206. Toscana, Poggioferro, 42°35′39″ N, 11°20′55″ E, 462 m, on *Quercus pubescens*, 5 June 2010, J. Nuñez-Zapata et al., MAF-Lich 17443, MAF-Lich 17447; SPAIN. Cadiz, 36°9′4.5″ N, 05°34′54.7″ W, 220 m, on *Quercus*, 19 October 2017 Crespo et al. coll. No. 7, DNA code 7197, coll. No. 8, DNA code 7198. Canary Islands, Tenerife, Degollada de Ten Alto, 28°20′37″ N, 15°51′36″ W, 829 m, on rock, 23 June 2009, A. Crespo et al., MAF-Lich 17242, La Escalona, 28°07′14″ N, 16°40′19″ W, 982m, on rock, 23 June 2009, A. Crespo et al., MAF-Lich 17256. Mallorca Municipio de Benyalbufar, Finca de Planicie, 39°40′15″ N, 02°30′34″ E, 460 m, on *Quercus ilex*,27 November 2009, A. Crespo MAF-Lich 17401, Son Ufanes, 39°48′05″ N, 02°57′59″ E, 156 m, on *Prunus*, 30 November 2009, A. Crespo et al., MAF-Lich 17406; TUNISIA. Gobernacion de Jendouba, 36°29′16″ N, 08°18′29″ E, 698 m, on *Quercus*, 30 March 2009, S. Castroviejo et al., MAF-Lich 17407.

## Figures and Tables

**Table 1:** Pairwise average values of Nei’s Gst and Hendrick’s G’st.

Nei’s Gst			
	*P. clandestina*	*P. mediterranea*	*P. tiliacea s. str.*
*P. mediterranea*	0.7389792		
*P. tiliacea* s. str.	0.7892928	0.7798357	
*P. pastillifera*	0.7938066	0.8331741	0.8652509
**Hedrick’s G’st**			
	***P. clandestina***	***P. mediterranea***	***P. tiliacea* s. str.**
***P. mediterranea***	0.9105523		
*P. tiliacea* s. str.	0.9452315	0.9314394	
*P. pastillifera*	0.9291603	0.9452874	0.9654279

**Table 2:** Analysis of Molecular Variance (AMOVA) for samples of *P. pastillifera*, *P. clandestina*, *P. mediterranea*, and *P. tiliacea* s. str.

AMOVA Components of Covariance	%
Variations between samples	94.906133
Variations within samples	5.093867
Total variations	100
Phi-samples-total = 0.9490613	

## Data Availability

The RAD sequence data used in this study were deposited in the NCBI Sequence Read Archive (SRA) through the accession number PRJNA1040836. Accession numbers for RADseq raw sequences are listed in Table S1. All the scripts that were used in this study can found in the Supplementary Materials.

## Supplementary Materials

The following supporting information can be downloaded at: https://www.mdpi.com/article/10.3390/jof9121180/s1. Table S1: data information of 95 *Parmelina* specimens utilized in this study. Data information includes sample name, name species, locality of collection, and herbarium numbers. File S1: phylogenomic and population structure analysis scripts used in this study.
