# Supplementary material for: Reference-Based Restriction-Site-Associated DNA Sequencing Data Are Useful for Species Delineation in a Recently Diverged Asexually Reproducing Species Complex (Parmeliaceae, Ascomycota)
Source: J Fungi (Basel). 2023 Dec 9;9(12):1180. doi: 10.3390/jof9121180 (PMC10744373; doi:10.3390/jof9121180)
Supplement: Supplementary file 1 [file jof-09-01180-s001.zip › File S1.pdf]

Phylogenomic analyses

#Model test

```
modeltest-ng -i ~/parmelina5/subdata2_outfiles/subdata2.snps -d nt -output
./modeltest.output -p 10 -a stamatakis -h g -T RAxML
```

#Raxml with BS

```
raxmlHPC -s ~/parmelina5/subdata2_outfiles/subdata2.snps -n parmelinap5 -m GTRCATX --
asc-corr=lewis -f a -x 3546564 -p 654683 -# 1000 -T 15
```

Analysis of population structure

#filtered vcf for all SNPs with a minor allele frequency (MAF) greater than or equal to 0.05 and a minimum coverage of 50%

```
vcftools --vcf ~/parmelina5/subdata4_outfiles/subdata4.vcf --max-missing 0.5 --maf 0.05 --out
Parmelina2.vcftools.vcf –recode
```

#Required packages

```
library(vcfR)
library(adegenet)
library(hierfstat)
library(qqman)
library(mmod)
```

#Loading vcf file into R genind object

```
vcf <- read.vcfR("~/Parmelina2.vcftools.vcf.recode.vcf")
data.genlight <- vcfR2genlight(vcf, n.cores = 10)
pop.file <- read.table('~/Parmelina2a.pop', header=F)
pop(data.genlight) <- pop.file[,2]
data.genind <- df2genind(as.data.frame(data.genlight), pop=pop(data.genlight), ploidy=1,
ind.names=indNames(data.genlight), loc.names=locNames(data.genlight))
data.genind
```

#Pairwise Fst

```
pwGstN <- pairwise_Gst_Nei(data.genind)
pwGstH <- pairwise_Gst_Hedrick(data.genind)
pwGstN
pwGstH
obj_seplocus <- seploc(data.genind);
```

#Calculation of Gst Nei

```
obj_pwGst <- lapply(obj_seplocus, pairwise_Gst_Nei);
obj_pairwiseGstnum <- sapply(obj_pwGst, as.numeric);
obj_pairwiseGstnum[obj_pairwiseGstnum<0] <- 0;
```

#Calculation of Gst Hedrick

```
obj_pwGstH <- lapply(obj_seplocus, pairwise_Gst_Hedrick);
obj_pairwiseGstHnum <- sapply(obj_pwGstH, as.numeric);
obj_pairwiseGstHnum[obj_pairwiseGstHnum<0] <- 0;
```

#DAPC

```
dapc1 <- dapc(data.genind, n.pca = 60, n.da = 2)
mycol <- c('red','darkblue','darkgreen','gold');
```

#print DAPC

```
scatter(dapc1, scree.pca = TRUE, posi.pca = "bottomleft", scree.da=TRUE,
posi.da="bottomright" , bg="white", pch=17:22,  cell=5, cstar=1, col=mycol, solid= 0.8, cex=1,
clab=0, leg=TRUE, txt.leg=paste("Clades",1:4))
compoplot(dapc1, posi='bottomright', ncol = 1, col = mycol, cleg = 0.8, cex.names = 0.4)
```

#Convert the .alleles matrix from ipyrad and exclude loci with less than 4 samples

```
python finerad_input.py --input ~/parmelina5/subdata4_outfiles/subdata4.alleles --minsample 4
```

#RADpainter. Calculate the co-ancestry matrix

```
./RADpainter paint ~/parmelina5/subdata4_outfiles/subdata4.alleles.min4.finerad
```

#fineStructure. Assign individuals to populations

```
./finestructure -x 100000 -y 100000 -z 1000
~/parmelina5/subdata4_outfiles/subdata4.alleles.min4._chunks.out
~/parmelina5/subdata4_outfiles/subdata4.alleles.min4.mcmc.xml
```

#fineStructure. Tree building

```
./finestructure -m T -x 10000 ~/parmelina5/subdata4_outfiles/subdata4.alleles.min4._chunks.out
~/parmelina5/subdata4_outfiles/subdata4.alleles.min4.mcmc.xml
~/parmelina5/subdata4_outfiles/subdata4.alleles.min4.mcmcTree.xml
```

#fineRADestructurePlot.R for visualization

#AMOVA-Required packages

```
library(vcfR)
library(adegenet)
library(poppr)
library(ape)
```

#**Read the data as a genind object**

```
vcf <- read.vcfR("~/Parmelina2.vcftools.vcf.recode.vcf")
data.genlight <- vcfR2genlight(vcf, n.cores = 10)
pop.file <- read.table('~/Parmelina2a.pop', header=F)
pop(data.genlight) <- pop.file[,2]
```

```
data.genind <- df2genind(as.data.frame(data.genlight), pop=pop(data.genlight), ploidy=1,
ind.names=indNames(data.genlight), loc.names=locNames(data.genlight))
data.genind

parmelinatable2 = read.table('~/parmelina1_strata.txt', header=T)
strata(data.genind) <- parmelinatable2
data.genind
```

#Read hierarchy file

```
hier(data.genind) <- ~Pop
data.genind
```

# AMOVA functions

```
parmelinamova = poppr.amova(data.genind, ~Pop)
parmelinamova
parmelinamova.test = randtest(parmelinamova, nrepet = 999)
str(parmelinamova.test)
```
