# Supplementary material for: Reference-Based Restriction-Site-Associated DNA Sequencing Data Are Useful for Species Delineation in a Recently Diverged Asexually Reproducing Species Complex (Parmeliaceae, Ascomycota)
Source: J Fungi (Basel). 2023 Dec 9;9(12):1180. doi: 10.3390/jof9121180 (PMC10744373; doi:10.3390/jof9121180)
Supplement: Supplementary file 1 [file jof-09-01180-s001.zip › Table S1.pdf]

Sample Name	Species	NCBI SRA accession
Pa_16634	Parmelina atricha: France: Pyrenees MAF-Lich 16634	SRR26887223
Pa_17559	Parmelina atricha: France: Pyrenees MAF-Lich 17559	SRR26887222
Pa_16633	Parmelina atricha: Spain: Gerona MAF Lich 16633	SRR26887211
Pa_17558	Parmelina atricha: Spain: Gerona MAF-Lich 17558	SRR26887200
Pa_4393	Parmelina atricha: Spain: Gerona, Coll. No. 6121C (DNA code 4393)	SRR26887189
Pc_4375	Parmelina carporrhizans: Spain: Canary is. Tenerife, Crespo et al. 14-3-2014, DNA code 4375	SRR26887178
Pc_3690	Parmelina carporrhizans: Spain: Canary is. Tenerife, Crespo et al. 23-6-2009, DNA code 3690	SRR26887167
Pc_4371	Parmelina carporrhizans: Spain: Canary is. Tenerife, Crespo et al. 14-3-2014, DNA code 4371	SRR26887156
Pc_4101	Parmelina carporrhizans: Morocco: Close to Beni-Hassan, Alors & Boluda. 21-10-2013, DNA code 4101	SRR26887145
Pc_2017	Parmelina carporrhizans: Spain: Cadiz, Crespo et al. 18-10-2017, Coll. No. 5	SRR26887134
Pp_17515	Parmelina pastillifera: Turkey: Canakkale, MAF-Lich 17515	SRR26887221
Pp_16471	Parmelina pastillifera: Turkey: Canakkale, MAF-Lich 16471	SRR26887220
Pp_20410	Parmelina pastillifera: Armenia: Tavush Prov., MAF-Lich 20410	SRR26887219
Pp_17507	Parmelina pastillifera: Germany: Bad Reichenhall, MAF-Lich 17507	SRR26887218
Pp_2015	Parmelina pastillifera: Spain: Caceres, Crespo et al. 23-11-2015	SRR26887217
Pp_17533	Parmelina pastillifera: UK: Wales, MAF-Lich 17533	SRR26887216
Pp_17532	Parmelina pastillifera: UK: Wales, MAF Lich 17532	SRR26887215
Pp_16473	Parmelina pastillifera: Spain: Cantabria. MAF-Lich 16473	SRR26887214
Pp_17508	Parmelina pastillifera: Germany: Bad Reichenhall, MAF-Lich 17508	SRR26887213
Pp_17547	Parmelina pastillifera: Norway: Nordaland, MAF-Lich 17547	SRR26887212
Pp_17541	Parmelina pastillifera: Norway: Nordaland, MAF-Lich 17541	SRR26887210
Pp_17540	Parmelina pastillifera: Norway: Nordaland, MAF-Lich 17540	SRR26887209
Pp_17545	Parmelina pastillifera: Norway: Nordaland, MAF-Lich 17545	SRR26887208
Pp_17544	Parmelina pastillifera: Norway: Nordaland, MAF-Lich 17544	SRR26887207
Pp_17482	Parmelina pastillifera: Italy: Friuli-Venezia, MAF-Lich 17482	SRR26887206
Pp_17465	Parmelina pastillifera: Italy: Friuli-Venezia, MAF-Lich 17465	SRR26887205
Pp_17464	Parmelina pastillifera: Italy: Friuli-Venezia, MAF Lich 17464	SRR26887204
Pp_17466	Parmelina pastillifera: Italy: Friuli-Venezia, MAF Lich 17466	SRR26887203
Pp_17469	Parmelina pastillifera: Italy: Friuli-Venezia, MAF Lich 17469	SRR26887202
Pp_17472	Parmelina pastillifera: Italy: Friuli-Venezia, MAF Lich 17472	SRR26887201
Pp_17471	Parmelina pastillifera: Italy: Friuli-Venezia, MAF-Lich 17471	SRR26887199
Pp_17470	Parmelina pastillifera: Italy: Friuli-Venezia, MAF-Lich 17470	SRR26887198
Pp_17468	Parmelina pastillifera: Italy: Friuli-Venezia, MAF-Lich 17468	SRR26887197
Pp_17473	Parmelina pastillifera: Italy: Friuli-Venezia, MAF-Lich 17473	SRR26887196
Pp_17451	Parmelina pastillifera: Italy: Toscana, MAF-Lich 17451	SRR26887195
Pp_17453	Parmelina pastillifera: Italy: Toscana, MAF-Lich 17453	SRR26887194
Pp_17454	Parmelina pastillifera: Italy: Toscana, MAF-Lich 17454	SRR26887193

Pp_17452	Parmelina pastillifera: Italy: Toscana, MAF-Lich 17452	SRR26887192
Pp_19537	Parmelina pastillifera: Portugal: Minho, MAF-Lich 19537	SRR26887191
Pp_19501	Parmelina pastillifera: Portugal: Minho, MAF-Lich 19501	SRR26887190
Pp_17386	Parmelina pastillifera: Spain: Navarra, MAF-Lich 17386	SRR26887188
Pp_17385	Parmelina pastillifera: Spain: Navarra, MAF-Lich 17385	SRR26887187
Pp_18341	Parmelina pastillifera: Spain: Navarra, MAF-Lich 18341	SRR26887186
Pp_17384	Parmelina pastillifera: Spain: Navarra, MAF-Lich 17384	SRR26887185
Pp_19654	Parmelina pastillifera: Austria: Oberosterreich, MAF-Lich 19654	SRR26887184
Pt_7197	Parmelina tiliacea: Spain: Cadiz, Crespo et al. 19-10-2017 coll. No. 7, DNA code 7197	SRR26887183
Pt_7198	Parmelina tiliacea: Spain: Cadiz, Crespo et al. 19-10-2017 coll. No. 8, DNA code 7198	SRR26887182
Pt_17531	Parmelina tiliacea: UK: Wales, MAF-Lich 17531	SRR26887181
Pt_17530	Parmelina tiliacea: UK: Wales, MAF-Lich 17530	SRR26887180
Pt_17538	Parmelina tiliacea: UK: Cumbria, MAF-Lich 17538	SRR26887179
Pt_17535	Parmelina tiliacea: UK: Cumbria, MAF-Lich 17535	SRR26887177
Pt_17526	Parmelina tiliacea: Turkey: Eskisehir MAF-Lich 17526	SRR26887176
Pt_17524	Parmelina tiliacea: Turkey: Eskisehir MAF-Lich 17524	SRR26887175
Pt_17522	Parmelina tiliacea: Turkey: Eskisehir MAF-Lich 17522	SRR26887174
Pt_19379	Parmelina tiliacea: Iran: Azarbaijan, MAF-Lich 19379	SRR26887173
Pt_19378	Parmelina tiliacea: Iran: Azarbaijan, MAF-Lich 19378	SRR26887172
Pt_17485	Parmelina tiliacea: Slovenia: Bled, MAF-Lich 17485	SRR26887171
Pt_17484	Parmelina tiliacea: Slovenia: Bled, MAF-Lich 17484	SRR26887170
Pt_17481	Parmelina tiliacea: Italy: Friuli-Venezia, MAF-Lich 17481	SRR26887169
Pt_17479	Parmelina tiliacea: Italy: Friuli-Venezia, MAF-Lich 17479	SRR26887168
Pt_17477	Parmelina tiliacea: Italy: Friuli-Venezia, MAF-Lich 17477	SRR26887166
Pt_19206	Parmelina tiliacea: Italy: Sicilia, MAF-Lich 19206	SRR26887165
Pt_17450	Parmelina tiliacea: Italy: Toscana, MAF-Lich 17450	SRR26887164
Pt_17447	Parmelina tiliacea: Italy: Toscana, MAF-Lich 17447	SRR26887163
Pt_17443	Parmelina tiliacea: Italy: Toscana, MAF-Lich 17443	SRR26887162
Pt_17304	Parmelina tiliacea: Morocco: Ifrane, MAF-Lich 17304	SRR26887161
Pt_17298	Parmelina tiliacea: Morocco: Ifrane, MAF-Lich 17298	SRR26887160
Pt_17303	Parmelina tiliacea: Morocco: Ifrane, MAF-Lich 17303	SRR26887159
Pt_17294	Parmelina tiliacea: Spain: Canary Is, Gran Canaria, MAF-Lich 17294	SRR26887158
Pt_17242	Parmelina tiliacea: Spain: Canary Is, Tenerife, MAF-Lich 17242	SRR26887157
Pt_17232	Parmelina tiliacea: Spain: Canary Is, La Palma, MAF-Lich 17232	SRR26887155
Pt_17256	Parmelina tiliacea: Spain: Canary Is, Tenerfie, MAF-Lich 17256	SRR26887154
Pt_17257	Parmelina tiliacea: Spain: Canary Is, Tenerfie, MAF-Lich 17257	SRR26887153
Pt_17340	Parmelina tiliacea: Spain: Caceres, MAF-Lich 17340	SRR26887152
Pt_17337	Parmelina tiliacea: Spain: Caceres, MAF-Lich 17337	SRR26887151
Pt_17553	Parmelina tiliacea: Sweden: Uppland, MAF-Lich 17553	SRR26887150

Pt_17551	Parmelina tiliacea: Sweden: Uppland, MAF-Lich 17551	SRR26887149
Pt_17550	Parmelina tiliacea: Sweden: Uppland, MAF-Lich 17550	SRR26887148
Pt_20278	Parmelina tiliacea: Armenia: Ararat Prov., MAF-Lich 20278	SRR26887147
Pt_20424	Parmelina tiliacea: Armenia: Tavush Prov., MAF-Lich 20424	SRR26887146
Pt_17497	Parmelina tiliacea: Austria: Salzburg, MAF-Lich 17497	SRR26887144
Pt_17495	Parmelina tiliacea: Austria: Salzburg, MAF-Lich 17495	SRR26887143
Pt_17494	Parmelina tiliacea: Austria: Salzburg, MAF-Lich 17494	SRR26887142
Pt_16485	Parmelina tiliacea: Germany: Bavaria, MAF-Lich 16485 (Epitypus)	SRR26887141
Pt_17512	Parmelina tiliacea: Germany: Bavaria, MAF-Lich 17512	SRR26887140
Pt_17511	Parmelina tiliacea: Germany: Bavaria, MAF-Lich 17511	SRR26887139
Pt_17510	Parmelina tiliacea: Germany: Bavaria, MAF-Lich 17510	SRR26887138
Pt_17504	Parmelina tiliacea: Germany: Bad Reichenhall, MAF-Lich 17504	SRR26887137
Pt_17501	Parmelina tiliacea: Germany: Bad Reichenhall, MAF-Lich 17501	SRR26887136
Pt_17407	Parmelina tiliacea: Tunisia: Gobernacion de Jendouba, MAF-Lich 17407	SRR26887135
Pt_17429	Parmelina tiliacea: Switzerland: Neuchatel, MAF-Lich 17429	SRR26887133
Pt_17427	Parmelina tiliacea: Switzerland: Neuchatel, MAF-Lich 17427	SRR26887132
Pt_17406	Parmelina tiliacea: Spain: Mallorca, MAF-Lich 17406	SRR26887131
Pt_17401	Parmelina tiliacea: Spain: Mallorca, MAF-Lich 17401	SRR26887130
Pt_17403	Parmelina tiliacea: Spain: Mallorca, MAF-Lich 17403	SRR26887129
